# A Comprehensive Review on Recent Advances in Plant Flour–Based Edible Tableware

**DOI:** 10.1155/ijbm/6206991

**Published:** 2025-08-06

**Authors:** Swarup Roy, Athira R. S. Pillai, Mitali Madhumita, Riya Joshi, Wanli Zhang, Shiv Shankar

**Affiliations:** ^1^Department of Food Technology and Nutrition, School of Agriculture, Lovely Professional University, Phagwara, Punjab 144411, India; ^2^Department of Food Technology, School of Health Sciences and Technology, University of Petroleum and Energy Studies, Bidholi, Dehradun, Uttarakhand 248007, India; ^3^School of Food Science and Engineering, Hainan University, Haikou 570228, China; ^4^Bioproducts Discovery & Development Centre, University of Guleph, Guelph, Ontario N1G 2W1, Canada

**Keywords:** biodegradable, edible cutlery, food, packaging, plastic waste, sustainable

## Abstract

Nowadays, plastic has become an integral part of our daily used products. Packaging is the sector where a significant portion of plastics are being used, and it has increased many folds after the recent pandemic. The plastic-based cutlery, cups, bowls, and plates have been commonly used in ready-to-eat packaged food, and they include mostly single-use plastic; thus, there is an urgent need for substitution with eco-friendly alternatives. The edible cups, bowls, and cutlery could be a promising alternative to the plastic counterparts. This review debated the current scenario in edible cutlery fabrication and characterization. The plant-based, eco-friendly edible flour materials are commonly used for fabricating edible cutlery such as bowls, cups, and spoons. The fortification and enrichment of additives into the edible cutlery and tableware were promising to improve the physical and functional performance. To develop edible cutlery, various flours such as millet, wheat, and rice have already been explored, and the results are promising for attaining sustainable development. The edible spoons prepared by using various flours such as finger millet and wheat flour with ashwagandha powder showed high proximate composition, including protein 5.96% and carbohydrates 85.73%. Similarly, the edible cutlery prepared using rice flour, wheat flour, and banana blossom powder resulted in a high water absorption capacity of 31.59% and showed high biodegradable capacity and decayed in 5 days. The use of this edible tableware not only reduces plastic waste issues but also makes our food healthier and nutrition-rich. Hence, this review aims to provide an overview of edible cutlery's needs and current status.

## 1. Introduction

Nonbiodegradable plastic manufacturing and use have grown significantly in recent years. Plastic manufacturing increased from 1.5 million metric tons in 2018 to 367 metric tons in 2020, according to the report, and is predicted to triple by 2050 [1]. In the end, Asia contributes 51% of the world's plastic production, followed by North America (18%), Europe (17%), and other continents [2]. Furthermore, just 9% of plastic garbage is properly recycled, while 22% is mismanaged. The globe is producing twice as much plastic waste as it did 20 years ago, and the majority of it ends up in landfills, is burned, or leaks into the environment [3, 4]. Additionally, the presence of plastic polymers in the environment causes defragmentation since many of the plastics that are now in use fracture into smaller pieces rather than degrading. Recent research has changed the term “microplastics,” which refers to the smaller bits, to “nanoplastics.” Numerous cattle and wild animals have intestines that contain these big and minute microplastics, which are then passed on to humans via the digestive tract [5, 6]. Furthermore, chemicals like plasticizers that are employed to increase the stability and functioning of plastics may also enter bodies of living things and build up in biological systems [7]. A variety of alternatives have entered our lives as a result of the growing use of single-use plastics and the general understanding of their harmful impacts. Action and international cooperation will be needed to reduce plastic pollution. This will include efforts to improve waste management and enhance recycling, as well as innovation, better product design, and the development of ecologically suitable alternatives. More than 120 nations have laws prohibiting and taxing single-use plastics, yet they are insufficient to lower pollution levels overall [8].

In actuality, the majority of rules only apply to products like plastic bags, which account for a very small portion of plastic trash and are more successful at preventing littering than reducing the use of plastics. Due to sedentary lifestyles, plastic utensils are one of the many plastic products that are discarded, seriously polluting the environment, particularly in urban and metropolitan areas. In actuality, consumers are more prone to discard cleaning items by using single-use cups, plates, silverware, and tableware [9]. Furthermore, since the recent COVID-19 outbreak, the use of single-use cutlery and tableware has grown. Nonedible cutlery is frequently used in street food, hotels, restaurants, and even in kitchens at home. When it comes to eating, cutlery is essential. According to reports, the kind, color, and form of the cutlery have an impact on how well a meal is enjoyed [10]. Synthetic, nonbiodegradable polymers produced from petroleum are used to make these cutlery items. Consequently, this plastic's disposal led to major issues and its eventual disposal in landfills or water bodies [11]. According to recent research, only about 40 billion pieces of cutlery are used annually in the USA, compared to over 120 million pieces in India and 640 billion pieces worldwide, and the number is growing quickly each year [12]. The global plastic-made cutlery market is large (∼10 billion dollars) and expected to double in the next decade [13].

The global disposal of used plastics has already led to widespread environmental pollution. Synthetic plastics take thousands of years to degrade, posing a serious threat to the environment. Over time, disposed of plastic breaks down into microplastics and nanosized plastics, which are extremely hazardous to the health of living organisms and the environment due to their toxic effects [14, 15]. Recent publications have highlighted the alarming health impacts of microplastics [16, 17]. Studies have shown that the chemicals found in microplastics are highly toxic, with some being mutagenic and carcinogenic [14]. The accumulation of microplastics in the food chain poses a significant threat to the health of living organisms, leading to potentially severe health issues [18–20]. There is a growing concern about reducing plastic consumption and increasing plastic recycling. Reports indicate that only 15% of discarded plastics are effectively recycled, contributing to environmental pollution [21, 22]. To address this escalating issue, researchers worldwide have proposed an alternative solution known as biodegradable and edible tableware, including edible plates, bowls, and cutlery [23–26].

Edible tableware has been around for a long time, dating back to the 1400s. The first bread bowl was created in 1427 to impress a British Duke. The Duke was so impressed that he provided funds to the Irish nobleman who invented it, enabling him to open a bread-bowl shop. In the 1930s, the Tostada bowl, made of stale tortilla, was introduced. This modern bowl is based on a Mesoamerican design and has been modified in various ways, including the mini Betty Crocker version. In the 1980s, the Sourdough boule bowl was introduced to promote San Francisco's clam chowder. This bowl became popular in the Bay Area and has been used by restaurants in the USA as a way to charge more for the soup.

Plant flour–based materials are substances obtained through the milling or grinding process of cereals, legumes, or tubers into fine powders or flours. These substances are primary raw materials for creating a variety of functional foods, including edible cutlery [23]. Plant-based edible cutlery allows meals to be served or consumed as a meal. Because the product is composed of a blend of grain flours, it is widely acknowledged to be organic, biodegradable, and eco-friendly [26, 27].

Edible cutlery has been sold commercially since 2010 by Bakeys, an Indian company [28]. Scientist Narayana Peesapaty of Hyderabad, India, saw people using khakhras instead of plastic spoons to scoop rice during a meal on an aircraft. He was motivated by it to fashion a flour spoon [29]. The two main justifications for using edible cutlery instead of plastic ones are biodegradability and environmental friendliness since plastic poses a serious risk to both the environment and human health.

Edible cutlery can be made using a variety of ingredients, but specific flours offer the desired properties for the final product. Ingredients such as millet flour, rice flour, rice bran flour, soy flour, sorghum flour, and others have their own nutritional benefits, which can improve the physical and chemical properties of the edible product when combined with other ingredients [22, 30].

These products are designed to be eaten after use or composted, offering a sustainable alternative to single-use plastics. Key features include being free from preservatives and synthetic additives, fully biodegradable, and safe for consumption, which helps reduce environmental pollution. Edible wares are increasingly used in food service, events, and as edible films or coatings to extend food shelf life and cut down on waste [27].

Plastic poses a significant threat to the ecosystem, making it crucial to replace plastic cutlery with better alternatives like edible cutlery [31]. The use of plastics and the challenge of their disposal are major issues in today's society [32, 33]. Consequently, plastics end up as microplastics in our bodies. Studies have indicated that plastics contain a harmful chemical known as bisphenol A. Bisphenol A, a chemical that mimics hormones, has been associated with various adverse health effects in children. These effects include altered behavior and obesity, reproductive abnormalities, changes in cardiovascular function, and cancer [34]. The edible cutlery is composed of calcium, potassium, vitamin B, vitamin A, and abundant fiber [35]. Whole grains like rice, lentils, millet, and wheat, which are the main components of food, promote health by preventing conditions such as obesity, constipation, coronary heart disease, and other diseases that pose a threat to health [36–38]. Additionally, incorporating natural bioactive ingredients such as hibiscus powder, mosambi peel, beetroot powder, and onion peel powder into the edible tableware (cups, bowls, plates, spoons, etc.) enhances their nutritional and functional value in addressing health issues [30, 37, 38]. However, challenges remain, such as achieving the necessary mechanical strength and barrier properties, ensuring shelf stability and food safety, managing higher production costs, gaining consumer acceptance, and meeting regulatory standards. Despite these hurdles, plant-based edible wares present a promising path toward more eco-friendly packaging and utensils [37].

The existing literature indicates that there have been limited comprehensive reviews on edible tableware up to this point. To our knowledge, only one review, conducted by Roy & Morya [26], has been published on edible cutlery, which focused on providing an overview of edible cutlery and its health benefits. In contrast, this review aims to present detailed and current information on the production and characterization of various types of biobased edible cutlery and tableware. Consequently, this review paper discusses the significance of edible cutlery, as well as its manufacturing, properties, and characterization, intending to offer a comprehensive understanding of the edible tableware currently in use. It is anticipated that the insights from this review will enhance consumer interest and awareness regarding the use of biobased edible cutlery. The systematic literature review was conducted using various scientific databases, including Google Scholar, ResearchGate, Elsevier's Science Direct, PubMed, and Springer Link, ensuring a comprehensive collection of relevant studies. Most of the selected articles span from 2015 to 2024, providing up-to-date insights into the field. The selection criteria focused on four key aspects: studies detailing edible tableware from plant flours, research providing fabrication and properties of edible plant flour–based edible tableware techniques, and papers discussing the application of plant-based edible tableware formulations. This structured approach ensured the inclusion of high-quality and relevant literature for the review.

## 2. Status, Importance, and Need of Edible Cutlery

Early humans used natural materials like stone and bones as primitive cutlery. Wooden spoons appeared in medieval England, serving as status symbols [24]. Metal cutlery became popular in the 18th century, with the elite preferring silver. Stainless steel later enabled widespread adoption of metal cutlery. After World War II, metal scarcity led to the emergence of plastic in kitchenware and cutlery. Plastic utensils were mass-produced in the 1960s as an affordable alternative to conventional cutlery. Plastic utensils were designed for single use, eliminating the need for cleaning. This disposability and low-cost increased demand in fast food and other industries. With fast food's rise in the 2000s, plastic cutlery pollution surged, tainting ecosystems. Plastics contain many carcinogenic chemicals like BPA, phthalates, and PVC that enter the human body as microplastics through the food chain [38]. Therefore, due to the urgent need for people's health and environmental protection, the development of safer and more sustainable cutlery is the focus of scientists at present. Edible cutlery is a natural, biodegradable alternative to plastic that could resolve disposable cutlery waste issues [9]. Taiwan's Sugu Company claims to have pioneered edible cutlery in 1986. While not yet widely available to consumers, surveys show demand for edible cutler [38]. A 2018 study by Patil & Sinhal identified factors driving this demand, including [24]:

1. Edible cutlery has a simple, functional design focused on utility rather than aesthetics.
2. Edible cutlery requires fewer resources since it is easy to make, use, and dispose of.
3. Pursuing alternatives like edible cutlery requires scientific innovation to increase the adoption of these novel, sustainable products.
4. Edible cutlery offers a cost-effective method to reduce ecological pollutants.
5. Edible cutlery's biodegradability and affordability provide a notable way to protect the environment while spreading nutrition, advancing food, nutrition, and ecological health.
6. Protecting against the health risks of petrochemical pollutants is crucial. Edible cutlery can advance this by significantly decreasing plastic waste.

In addition to the above reasons, edible cutlery itself can also be used as a kind of food, which can supplement the human body's dietary balance through dietary matching by loading some nutrients into edible cutlery. For example, edible utensils for children's diet can attract children's interest through appearance and then supplement with regular food for nutrition.

Research shows that biodegradable, compostable, and edible products are a promising replacement for plastic serving wares. A market research report predicts substantial growth in edible cutlery, with the global market valued at $24.86 million in 2018. Experts forecast the market to reach $56.97 million by 2026, demonstrating the increasing demand for sustainable and eco-friendly dining options [39]. The most similar concept of edible cutlery is edible packaging, the main raw materials of which are similar, are composed of some edible polymer macromolecules, and are in direct contact with food and can be eaten directly as a part of food. Edible food packaging has been used commercially in various parts of the world in recent years. For example, in Asia, edible rice packaging is used for candies. Bangladesh utilizes 250 tons of plastic cutlery monthly across industries, prompting research into biodegradable options using rice, wheat, and sorghum. To reduce plastic waste, Indonesian company Evoware is developing seaweed packaging for food items. Malaysia also generates substantial plastic waste, and the food industry demands alternatives after plastic bans. Studies in Malaysia have molded robust edible cutlery using rice, sorghum, and wheat flour blends, ideal for soups and frozen desserts [40, 41]. Earlier report shows biscuit dough has the longest shelf life for edible cutlery, while seaweed and agar are more fragile [42]. Japan's Koratt Bakery and Orto Cafe make edible plates and utensils from biscuit dough *via* Rice-Design. Honest in Yokohama uses seaweed cups. Poland's Biotrem has wheat bran cutlery and distributes it globally. Notpla in London created Ooho, an edible seaweed capsule as a biodegradable alternative to plastic cups. In the United States, Loliware produces flavored seaweed straws now used by major companies, aiming for 30 billion produced by 2020. The USDA developed an edible milk protein film to replace plastic cheese wraps. Mexico's E6PR makes biodegradable wheat and barley containers for beverages to avoid ocean plastic pollution. South Africa's Munch Bowls are vegan, edible wheat bowls. France's Poilane makes edible cracker cookie utensils. Global efforts continue to reduce plastic cutlery usage [9, 38]. India disposes of around 120 billion plastic cutlery pieces annually with widespread usage and casual disposal. Edible cutlery is still emerging, pioneered by Bakeys in 2010, now producing 50,000 units daily with 25 million global orders by 2016. Research into nutritious, biodegradable edible cutlery in India is still nascent but promising, using materials like Moringa oleifera husk for compostable variants. Further development of eco-friendly, nutritious edible options can help mitigate India's substantial plastic waste and environmental impact [38, 43]. Therefore, edible cutlery is the focus of attention in the field of food science and the food industry in the future. In addition, it is worth mentioning that for future cost reduction and sustainable development of edible cutlery, some natural materials derived from agricultural waste are considered to be excellent candidates for the manufacture of edible cutlery.

Agrofood industries produce large quantities of waste, primarily pomace and bran from agricultural and food processing operations. This waste is often used as animal feed, but new uses are being explored. There is growing interest in utilizing agri-food byproducts like fruit pomace and cereal brans to produce sustainable biodegradable products such as edible cutlery objects. For example, apple pomace contains compounds that can be used to make bioplastics or molded into 3D printed items. Converting abundant agrofood waste into biodegradable consumer goods presents an opportunity to reduce waste and develop eco-friendly products [44, 45]. For example, a recent study tested the feasibility of producing biodegradable disposable paper cups coated with beeswax from pineapple peel, orange peel, and Mauritian cannabis leaves. The results showed that the disposable cups produced from hemp and jackfruit showed good mechanical properties and appearance structures, and were sufficient to retain cold water for 30 min (minimum) without any leakage [46]. Although the necessity of the development of a lot of edible cutleries has been mentioned above, there are some limitations in its development [26]. Edible cutlery is often composed of some biological macromolecular polymers, so the cutlery itself may have problems such as microbial contamination [38]. Therefore, edible cutlery can also be equipped with some natural antibacterial agents or antioxidants, which can improve the shelf life of the edible cutlery itself. In addition, the main challenges for the industrial production of edible cutlery include its production methods and performance enhancement strategies. For example, disposable cutlery often needs to meet the mechanical properties and water resistance of practical applications, and most edible natural polymers are hydrophilic, so it is necessary to enhance the performance of edible cutlery through some green methods, rather than through the addition of chemical synthesis. Therefore, the fabrication and characterization of recently developed edible tableware has been discussed briefly in the following section.

## 3. Fabrication and Characterization Process of Edible Tableware

**Figure 1:**
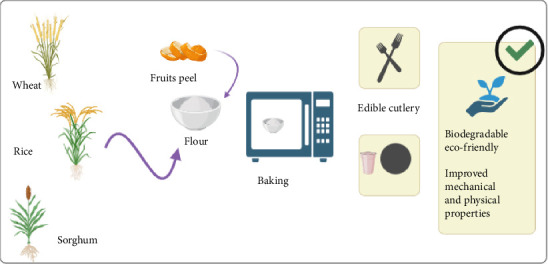
Fabrication process of edible bowls and cutlery.

Nowadays, the edible, biobased, and sustainable cutlery has gained a lot of interest from the researcher. Natural edible cutlery can be replaced with synthetic cutlery in the food industry extensively, contributing significantly to the green economy and environment [47, 48]. Biodegradable cutlery is in demand today as substitute for plastic cutlery and reducing the disposable waste [23]. From most of the recent studies, it is found that cutlery items having good biodegradability are not only delicious, healthy, and environmentally friendly but also decrease the release of chemically toxic compounds (Figure 1). The recent advancement on the biobased edible cutlery and tableware is comprehensively discussed and presented in Table 1.

Plant-based flours, made from sources like cereals, legumes, seeds, and tubers, are widely utilized in making edible cutlery because they are sustainable, nutritious, and capable of forming durable structures. Common examples include wheat, rice, millet, sorghum, chickpea, and corn flours [59]. These flours support the cutlery, helping it maintain its shape during use. The natural starches in the flours act as binding agents when exposed to heat, enhancing the stability of the product. Each type of flour also contributes distinct flavors and health benefits. For example, wheat flour offers elasticity, rice flour adds a crisp, gluten-free texture, and flours like millet, sorghum, and chickpea are rich in fiber and protein. Often, different flours are combined to improve the overall taste, texture, and strength of edible tableware [59].

Wheat flour is extensively used in edible tableware due to its textural and sensory properties. The flour can be blended with a small proportion of maize flour, and wheat flour-based tableware exhibits good organoleptic qualities, strength, and overall acceptability. Another advantage is that it is also cost-effective compared to other products in the market [49]. Wheat flour is rich in protein, carbohydrates, and energy, making it a nutritious choice. However, it can result in heavier, denser products and the natural oil present in it may spoil the product more quickly. Some individuals might also find the taste too strong or experience digestive discomfort. Despite these drawbacks, wheat flour remains a popular and practical base for biodegradable tableware, supporting green marketing and environmental sustainability [60].

Rice flour is another commonly used flour for plant-based tableware, valued for its neutral flavor and hypoallergenic properties. It demonstrates lighter and more delicate products compared to wheat flour and is mainly suitable for gluten-free products. Its fine texture allows for smooth finishes in molded tableware. However, rice flour–based items may lack the elasticity and strength of wheat-based products, making them more prone to breakage, particularly when exposed to heat or moisture. Additionally, rice flour has a lower protein content, which can affect the structural integrity of the final product [61].

Sorghum flour is recognized for its high fiber content and ability to add nutritional value to edible tableware. It is gluten-free, making it suitable for people with celiac or gluten intolerance. Sorghum flour also imparts a mild, earthy flavor and can improve the antioxidant profile of tableware [62]. On the other hand, sorghum-based products may be more brittle and less cohesive than those made with wheat or rice flour. Binding agents are added to achieve the right texture and strength, which often requires blending sorghum with other flours [51].

Banana flour, made from green, unripe bananas, is rich in resistant starch and offers a unique, slightly sweet taste to edible tableware. It is naturally gluten-free and can enhance the nutritional profile by adding dietary fiber and micronutrients. Banana flour is also known for its water retention, which can help keep tableware from becoming too dry or brittle. However, its distinct flavor may not suit all applications, and the high starch content can sometimes lead to a gummy or chewy texture if not properly balanced with other ingredients. Additionally, banana flour can be more expensive and less widely available than staple flours like wheat or rice [52, 53].

Recently, a few reports have been published on the development of biobased edible cutlery and tableware. Choeybundit et al. [38] prepared some cutlery items by taking *Morning magnificence* stems (MGS) and gathered it as new vegetable waste from a neighborhood ranchers' market, Chiang Rai, Thailand. MGS were washed utilizing refined water to eliminate the residue and soil particles. Washed MGS were cut into pieces 10 mm long with a treated steel scissor and dried at 80°C for 15 h in a hot air plate dryer. To get crude morning glory stem fiber (MGSF) powder, dried MGS were ground in a grinder and sieved with a stainless steel 230 mesh (63 m pore size). When not in use, the powdered fibers and MGSF were transferred into zip-lock bags and dried in a desiccator at 25°C. Secondly, the same author made a biocomposite arrangement from soy protein isolate (SPI) without and with MGSF, and the SPI and unrefined MGSF were dried out in the broiler at 60°C and 80°C for 6 and 15 h before the planning of the biocomposite, separately. SPI cutlery was ready by blending 70% of SPI and 30% glycerol with various degrees of MGSF (5%, 10%, 20%, w/w) to get a final weight of 8 g for each cutlery test. The further characteristics of SPI are separated from soybeans as a plant result that shows incredible biodegradable and compostable properties compared with the plastic material. SPI has been utilized as a vital material for advancing biodegradable composites as a plastic replacer. A few changes of SPI have shown promising results to work on mechanical and warm properties. Various items obtained from soybeans include defatted soy flour, soy protein concentrate, and SPI sum 50%, 70%, and 90% of protein content, individually. The resulting insight of this work is appealing and could be useful to fabricate the eco-friendly cutlery items. In this way, SPI built up the MGSF composite that can be used as eco-accommodating and could be financially planned into consumable and biodegradable cutlery for food applications.

In another work, Nehra et al. [12] have developed a biobased edible bowl by using different proportions of ingredients like finger millet flour, refined flour, jaggery, and xanthan gum along with brewer's spent grain (BSG) which shows better physicochemical properties. The author described that the combination of millet and refined flour was effective to make a high-quality edible bowl. The addition of BSG into the edible cutlery makes it harder and better in terms of oil and water absorption. The edible bowl showed good biodegradability in the soil burial test. The BSG-fortified edible bowl also showed superior antioxidant potential compared to the control. The antioxidant activity of the edible bowl is dependent on the content of BSG. Thus, the BSG-fortified plant-based biodegradable edible bowl could be a potential alternative to a commercial plastic bowl. Therefore, the study concluded that biobased ingredients can be molded into a bowl easily which can be a suitable alternative to the plastic-based bowl. Moreover, Banh et al. [54] developed and optimized an edible spoon by using the Taguchi method. The edible spoon was also analyzed by conducting different tests by showing a greater durability and good appearance.

Some of the manufacturing industry also developed some edible tableware like edible soup bowls and cups by using wheat bran along with the addition of fat, emulsifier, preservatives, and milk products having no hazardous chemical content. Moreover, some of the companies made Bakeys edible cutlery like spoon and baked biscuit edible cups and bowls by using rice, wheat, and millets by adding different flavors such as coconut curry, wasabi sesame, gingerbread, cranberry, and cornbread. Edible baked spoons are also prepared by Dordevic et al. [55], by using different percentages of grape seed flour, proso millet, wheat, xanthan, palm oil, and water. The addition of grape seed flours distinctly affects the color of the spoon as it changes from black to yellowish-white due to the pale-yellow color of the powder. Moreover, the functional properties of the spoon immensely increased due to the strong antioxidant action of the grape seed [55, 60]. The antioxidant edible cutlery is beneficial for health as well, which could be effective in preventing oxidation of protein present in plant-based flour. Evoware [63] also prepared edible-colored cups prepared by seaweed using different flavors like coffee, vanilla, strawberry, and peppermint. Mukherjee and Raju in 2023 fabricated edible cutleries by taking composite flour by taking whole wheat, foxtail millet, and roasted Bengal gram along with other ingredients having different percentages like skimmed milk powder, orange fruit powder, and beetroot extract [35]. The daily intake properties, phytochemical analysis, functional properties, and economic cost of the prepared edible cutleries are analyzed. The cost of the developed materials was estimated to be 2 rupees per piece which is affordable to the consumers. Moreover, authors have stated that the cost of the plant-based cutlery was little cheaper than the commercially available biobased cutlery made by Trishula incredibles, Gujarat, and Ediwares, Haryana. Nishihara and Kakehi fabricated some edible cutleries by taking rice flour and starch syrup [50]. The materials are prepared in the shape of cutleries and baked them at different baking times, i.e., for edible fork (baking time 17 min) and for edible spoon (baking time 15 min). Both the baked edible items are bent upward and downward to reduce the curvature. The materials are fabricated in different shapes and sizes by using extrusion-based printing. Siddiqui et al. also developed edible spoons by using different percentages of mosambi peel and sago powder, gum Arabic, and gelation with the use of experimental design [37]. The prepared dough is shaped to spoon with the help of the required mold and dried in a hot air oven at 80°C for 2 h. Then, the molded spoon is packed in HDPE bags and stored at 8°C. The physicochemical properties such as moisture content, ash content, fat content, protein content, water absorption capacity, color test, organoleptic properties, and biodegradability test are analyzed.

Rajendran et al. prepared a nutritious cutlery item by using wheat flour, pearl millet, barnyard millet, salt, and water [49]. The dough prepared by dough sheeter was cut into spoon shape using steel spoon punch and baked at 160°C for 45 min to maintain a desired texture and crispness. The spoon is prepared at an optimum condition of 50.12% wheat flour, 26.18% barnyard, and 0% pearl millet. The parameters like water absorption percentage, proximate analysis, and texture profile analysis of the prepared cutlery were analyzed. The effect of composition of the cutlery on water absorption was analyzed at room temperature (29°C), cold temperature (10°C), and hot temperature (50°C). Moreover, the parameters like hardness, fracture ability, springiness, cohesiveness, gumminess, chewiness, and resilience are analyzed.

Recently, Iqbal et al. developed some cutlery items (spoon, fork, and bowl) by taking different percentages of rice, sorghum, and millet flour with other ingredients and analyzed its functional properties [64]. Water holding capacity and oil holding capacity are found to be highest, i.e., 47.70 ± 1.120 and 135.16 ± 2.31, respectively, in Blend A (40 g of rice flour, 30 g of sorghum flour, and 30 g of wheat flour) compared to Sample B and Sample C. The Blend A sample also showed highest solvent retention capacity, high tensile strength, and overall acceptable sensory values along with good proximate values. All the cutlery items are also buried into soil to check the biodegradability test, and the Blend A sample is degraded within 5–7 days. Kabir and Hamildon also prepared edible spoon by taking different percentages of flours (rice, wheat, and sorghum) [58]. The prepared dough made from different flours is pressed on the steel spoon and baked in an oven at 300°C–360°C for 10–15 min. Water absorption percentage is high in Sample 1 (150 g of wheat flour and 100 g of rice and sorghum flour) because of having more volume of wheat flour.

Thagunna et al. prepared biodegradable edible items (bowl and spoon) by taking different percentages of rice flour, millet flour, wheat flour, jaggery, salt, and banana blossom pastes and the prepared dough was placed on a steel spoon and cooking bowl and baked at 180°C for 40 min [57]. The characteristic parameters like proximate composition (moisture content, protein, fat, fiber, and ash), water absorption capacity, and sensory analysis were analyzed in this study. Among all the samples, Sample 3 having 40 g of rice flour, 20 g of millet flour, 30 g of wheat flour, 10 g of banana blossom powder, 15 g of jaggery, 1.5 g of oil, and 0.35 g of salt showed good results. In Sample 1, carbohydrate content (90.43%) was found to be higher, whereas Sample 1 showed good sensory attributes using a 9-point hedonic scale and high water absorption capacity (31.59%). Based on the biodegradability test, all the samples showed a good result and decayed within 4–5 days. Different edible films are prepared by taking different percentages of moringa leaves powder, wheat flour, foxtail millet, and finger millet with the addition of different binding agents like egg, corn starch, gelatin, and rice flour [65]. The prepared dough is shaped into an edible spoon by baking at 65°C for 1 h. The proximate composition and drying characteristics (moisture content, drying rate, and moisture ratio) of the prepared edible spoon were also analyzed.

In another report, Hazra and Sontakke developed edible spoon by using different flours (finger millet, sorghum, and wheat flour) and ashwagandha powder with a ratio of 17:20:60:03 [31]. The prepared dough was made into edible spoon with a baking temperature of 100°C–150°C for 20–25 min. Among the proximate composition, protein and carbohydrate percentage of sample containing without ginseng powder were found to be higher, i.e., 5.96% ± 0.025 and 85.73 ± 0.05, respectively. However, the sample containing 4% ginseng powder showed highest percentage of ash, fat, and crude fiber. Water absorption capacity of all samples was also analyzed from 5 to 30 min, and it is increased gradually. Moreover, the presence of Indian ginseng in the plant-based edible cutlery exhibited strong antioxidant activity and the activity is dependent on the content of ashwagandha powder. The addition of ashwagandha into the edible cutlery could highly beneficial for the health owing to the medicinal properties. All the samples showed good results on soil burial test and degraded within 4 days. Moreover, the fracture ability and hardness of all edible spoon were also analyzed and found to be high in the sample containing 3% ginseng powder [31].

Sindhu et al. also prepared different colored edible spoons by using different natural color extracts (beetroot, spinach, and jamun) [66]. The edible-colored spoons are prepared by using different percentages of sorghum flour, wheat, and rice flour with the addition of sorbic acid and guar gum. The prepared dough was kneaded and filled in a spoon mold and baked at 160°C for 2 h in a hot air oven to make an edible spoon. The physicochemical analysis (moisture content, ash content, protein content, and fat content), texture analysis, color analysis, and soil burial test of all the developed samples were analyzed. Ash, protein, and fat percentage are found to be higher in beetroot-enriched edible spoon, whereas the hardness is high in jamun-enriched edible spoon. All the colored edible spoon showed a good result on soil burial test by decaying within 5–7 days.

**Figure 2:**
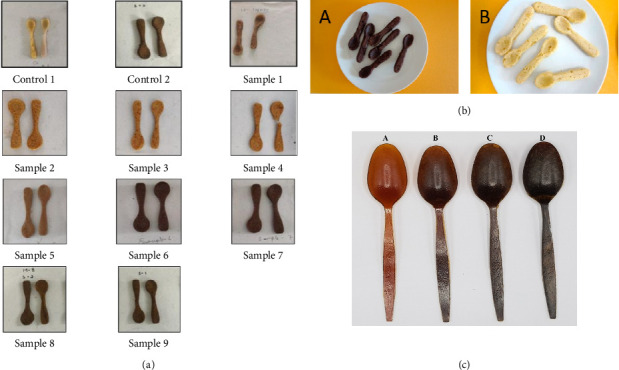
The specimens of biodegradable spoons using various types of ingredients. (a) Biodegradable spoons with various combinations of sago and mosambi peel powder (adapted from Siddiqui et al. [37], reprinted from the Royal Society of Chemistry, no permission needed); (b) the examples of biodegradable spoons with (A) and without (B) grape seed flour (adapted from Dordevic et al. [55], no permission needed); (c) soy protein isolate–based cutlery supplemented with different levels of morning glory stem fiber (0%, 5%, 10%, and 20%) (adapted from Choeybundit et al. [38], reprinted with permission, Elsevier publisher).

Sazeli et al. also prepared biodegradable cup which can replace the plastic, glass, and stainless-steel cutleries [42]. The biodegradable cup is prepared by taking corn and barley flour (7 parts) with water (3 parts). The characteristic parameters such as quality performance and long-lasting period of the developed biodegradable cup are also analyzed and resulted good results. In another recent report, Gupta et al. prepared biodegradable cup and bowls by using waste materials of paddy straw and pine needles [22]. Authors have added different binding agents such as gum acacia, potato starch, and carboxymethyl cellulose to the paddy straw and pine needles pulp to develop the biodegradable cup and bowls. To improve the hydrophobicity, the edible cups and bowls were further coated with polylactic acid (PLA). The different characterizations, such as water and oil absorption, were tested which indicates the good physical properties. Moreover, this report suggests 100% biodegradability of the cup and bowls prepared using pulp of pine needles and paddy straw within the 5 weeks.  Figure 2 shows the pictorial image of various types of biobased fabricated edible spoons fabricated by various researcher recently. It can be seen from Figure 2 that the digital image of the biobased spoon is comparable to the commercially available plastic-based spoon, but there is scope for improvement.

## 4. Production Cycle of Edible Tableware

The production cycle of edible cutlery begins with the appropriate selection of raw materials such as rice, wheat, millet, or sorghum flours. These materials are mixed with water or any other natural binding agents, such as guar gum or xanthan gum to create cohesive dough. Based on whether the cutlery needs to be sweet, savory, or colors, natural flavors, salt, and sweeteners can also be added. The ingredients are kneaded to form a uniform dough with the ideal consistency, which is both pliable and firm enough to retain its shape during processing. The resulting dough is fed into specialized molds to get utensil shapes like spoons, plates, and knives and forks [53].

**Figure 3:**
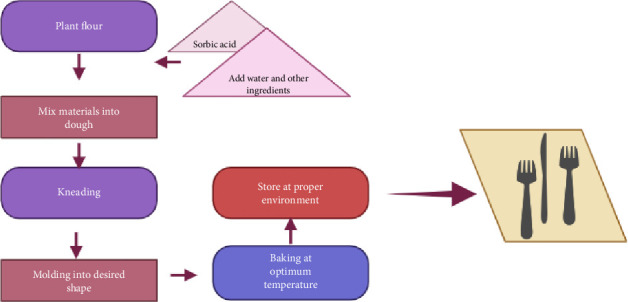
Illustration of the production cycle of edible tableware.

The molding process typically utilizes industrial machines or hydraulic presses that apply sufficient pressure to form and retain the intended utensil shape. The shaped dough is baked under high temperatures, typically ranging from 180°C to 250°C [31]. This step is considered as crucial for ensuring the cutlery becomes crunchy, rigid, and durable enough for practical use. In certain manufacturing processes, hot air drying or dehydration techniques are practiced as an alternative to baking, effectively removing moisture while preserving both the flavor and structural integrity of the cutlery. Once the baking or drying is complete, the cutlery is cooled under controlled temperature conditions to prevent moisture development and maintain its firmness. Subsequently, the cutlery is subjected to diligent quality control inspections to ensure it is free from defects such as deformities or cracks and meets established standards for texture, taste, hygiene, and overall quality [66–70]. The illustration of the production cycle is schematically represented in Figure 3.

## 5. Properties of Edible Tableware

**Figure 4:**
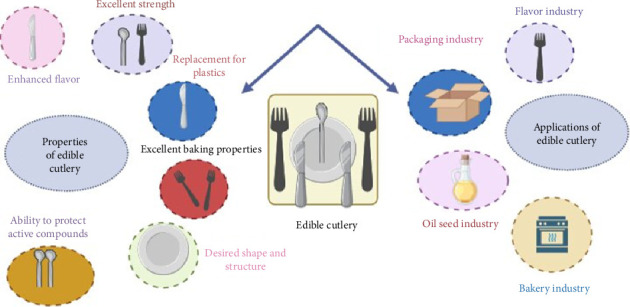
Properties and applications of edible tableware.

The properties of the edible cutlery and tableware depend on many factors such as the ingredient, fabrication process, and baking temperature and time. Among the different factors, the selection of proper or optimum combination of the biobased flour is most crucial to make edible tableware and cutlery (Figure 4). On the other hand, Table 2 summarizes the composition, mechanical properties, and biodegradability of different plant-based flours.

Choeybundit et al. developed an edible and biodegradable cutlery using the hydraulic hot press molding method by taking the ingredients like SPI and crude MGSF [38]. The characterization of cutlery samples with MGSF and without MGSF such as color and physicochemical properties along with the soil burial test is analyzed. The baked edible spoons prepared by Dordevic et al. have good nutritional value and mechanical properties [55]. The baking temperature of the process has a good impact on the edible spoon that increased textural properties of the materials to maintain the sustainability. The edible cutleries can be consumed with the food products by giving special attention to the physical and mechanical parameters like resistance to leakage, strength on flexural properties, biological and chemical safety point of view, way of producing, packaging, and transporting. The detailed properties of the developed edible cutlery prepared by Mukherjee and Raju analyzed and resulted that the composition having 35 g of foxtail millet flour, 35 g of whole wheat flour, 20 g of roasted Bengal gram flour, 5 g of skimmed milk powder, 2.5 g of orange powder, and 2.5 g of beetroot extract showed good properties and meets the RDA [35]. The composition blend also showed good structural, functional, and sensory properties. The phytochemicals like zeaxanthin, saponin, catechin, betacyanin, hesperidin, lignan, glutathione peroxidase, alkyl resorcinol, and ferulic acid are found among which the lignan compound showed maximum, i.e., 7.94 mg/kg.

Siddiqui et al. recently analyzed the physicochemical properties of all developed edible spoons having different percentages [37]. Among all the samples, S5 (mosambi peel, 50%, and sago starch, 50%) showed good results having the lowest moisture content, i.e., 6.67%, and fat content, i.e., 4.96%. However, in S6 sample, having the percentages of mosambi peel (60%) and sago starch (40%), the protein content was high, i.e., 2.33%. Moreover, S2 sample (mosambi peel, 20%, and sago starch, 80%) showed a good water absorption test having enhanced durability. S3 sample (sago starch, 70%, and mosambi peel, 30%) showed highest color values, whereas S8 sample showed lowest values and S5 sample (mosambi peel, 50%, and sago starch, 50%) got the highest overall acceptability rate. All the samples showed good biodegradability test among which S5 showed a good biodegradability test, i.e., 64.13%. Overall, S5 sample having 50% sago starch and 50% mosambi peel showed better results compared to other samples in aspects of all characterizations.

Gupta et al. fabricated plant waste pulp-based cup and bowls by using waste materials of paddy straw, pine needles, and different binding agent (potato starch, carboxymethyl cellulose, and gum acacia), and they further coated it with PLA [22]. The mechanical properties (tensile strength, burst index, tear index, and bending stiffness) of the PLA-coated bowls and cups were better than uncoated counterparts. Furthermore, the PLA-coated cups and bowls showed good antimicrobial activity toward *S. aureus* and *P aeruginosa*. Kumar et al. conducted study on various materials and their properties for the development of edible tablewares. Based on the research, wheat straw is found to be a suitable material for the edible plate (2.5 mm thickness) for livestock, and wheat bran can be utilized for the preparation of edible cutlery for humans. The nutritious components present in the straw can be excellent feed for livestock; additionally, it promotes the utilization of agricultural waste through sustainable methods [23].

Another work by Sazeli et al. discovered that corn and barley cutlery have shown better characteristics compared to plastic products. It has been recorded that the temperature endurance is 200°C for 20 min and it takes 27 days for the complete degradation where plastic commodities take 1000 years for degradation. Based on the quality evaluation, the edible cutlery has excellent water-holding capacity (22 min) and can bear a weight less than 12 kg before breaking. The estimated price of a single piece is 0.25%, which can be afforded by the consumers [42]. Buxoo and Jeetah utilized the fibers extracted from the fruit peel wastes (orange and pineapple) and Mauritian hemp leaves which are commonly found as feedstock, and agrowaste for landfilling was used for the production of biodegradable cups. The prepared cups exhibited excellent mechanical properties, contributed by the addition of fruit peels, particularly pineapple peel. The ratio of Mauritian hemp leaves and pineapple peel at 40:60, and high cellulose content in the peel, lower lignin content, and stronger interfacial bond attributed to high tensile strength of the product. Furthermore, the coating of beeswax (natural additive) at 0.7 mm thickness prevented the water leakage [46].

Various nations are also encouraging edible cutlery one classic example of edible cutlery if from Bakeys, it was established in 2011 by a groundwater specialist, Narayana Peesapaty to deliver consumable cutlery as a substitution for the plastic cutlery. Plastics are poisonous and cancer-causing, and they can siphon into the food. The natural cutlery items (forks, chopsticks, and even forks) can be made from dried millets (sorghum or jowar), rice, and wheat. If the chopsticks and spoons are placed in food and water, neither will become soiled. They are easy to eat at the end of a meal and only soften after 10–15 min. Regardless of whether disposed of, they disintegrate inside 5–6 days since they are biodegradable.

International Crop Research Institute for Semi-Arid Topics (ICRISAT) in Hyderabad also prepared edible cutlery by taking jowar and sorghum which serves to balance out the degrees of groundwater, and utilization of jowar for the palatable cutlery creation. It is the challenge of raising awareness about the negative effects plastic has on health. The developed edible cutlery is completely vegan, free of trans fat, dairy, and preservatives. The Bakeys can produce 100 sorghum-based spoons, and in correlation with corn, it produces 50 spoons. The energy costs are limited through a self-loader process that limits squander and amplifies productivity. Also, the low water uses for each spoon (under 2% of the weight per spoon) and permits the spoons to have an extremely lengthy timeframe of realistic usability of 2 years while keeping up with their firmness. In India, Bakeys now sells 1.5 million spoons annually. The flavors they use are as follows: ginger–cinnamon, ginger–garlic, sugar, celery, cumin, dark pepper, mint–ginger, carrot, beetroot, etc. By incorporating different natural flavors, spices, colorant in cereals, pulses, and millet flour to make different flavored, colored edible cutleries were tested which can improve the nutritional properties, antioxidant, and antimicrobial properties with an enhancement of sensory properties. In the future, the value-added nutritious components can be incorporated along with natural pigments or flavoring substances to the biodegradable edible cutleries, tableware to enhance the taste and shelf stability.

Fortification and encapsulation are two important techniques used to incorporate active compounds into edible cutlery, each with its distinct purposes and providing several advantages. Fortification involves the addition of nutrients or bioactive compounds, such as vitamins, minerals, polyphenols, antimicrobials, or antioxidants, to the cutlery to increase its nutritional value and provide added health benefits such as anticancer, anti-inflammatory, and antidiabetic. This can make the product more appealing to health-conscious consumers and offer a marketing advantage as a value-added item as well. However, fortification can also cause challenges, such as potential alterations in taste and texture, stability issues with certain nutrients over time, and the need to comply with food safety and labeling regulations, potentially making it less appealing to the health-conscious consumers [12, 78]. There is also the issue of allergens, as some functional ingredients might trigger allergic reactions in certain individuals, necessitating thorough labeling and caution in this regard. Regulatory compliance is another hurdle, as adding functional ingredients may require additional approvals and adherence to food safety standards, complicating the manufacturing, increasing cost, and also the distribution process. Lastly, consumer perception plays a significant role, as people have different preferences and concerns about functional ingredients in edible products, which can influence market acceptance and adoption. These considerations underscore the importance of balancing innovation with practicality and consumer satisfaction when developing functional edible cutlery [22].

On the other hand, encapsulation implies enfolding functional compounds within a carrier material, protecting them from environmental factors like moisture, light, and oxygen, which helps to conserve their stability and extend shelf life. Encapsulation also allows for the controlled release of these compounds and can mask any unpleasant tastes or odors, improving the overall sensory experience. Regardless of these benefits, encapsulation can add complexity and cost to the manufacturing process, which requires careful selection of safe and effective encapsulating materials, expected to increase the overall cost and may necessitate additional regulatory approvals. Together, these techniques will enhance the nutritional profile and functionality of edible cutlery, though they also introduce certain challenges that must be managed during product development and production. But so far not much studies have been reported on encapsulated functional compound activated edible cutlery.

## 6. Life Cycle Assessment (LCA) of Edible Cutlery

LCA is a systematic and comprehensive tool used to evaluate environmental impact throughout the life cycle. LCA helps to analyze the contribution of each stage, particular impacts, and reveals potential shifting of environmental burden between different products or services providing the same functional purposes [79, 80]. Anand et al. [81] conducted LCA and littering indicator against the environmental effects of edible cups and reusable cups. Across the most significant environmental impact categories, edible cups exhibited the highest burden, while reusable cups had the least impact. Based on the standard assumption, the environmental impact per cup use ranges is 0.004 to 0.1 kg CO_2_ equivalent, and the eutrophication impact ranged from 6.26 × 10^−6^ to 4.21 × 10^−4^ kg·N [59]. However, the sustainability of edible cutlery is based on multiple factors such as raw materials used, proper composition, and renewable energy in production and can be a viable alternative for single-use plastics when implemented thoughtfully within a proper waste management system [79].

## 7. Economic Feasibility of Edible Tableware

Plant-based edible and biodegradable tableware are advantageous over the synthetic plastic-based counterpart [12, 80]. Even though there is potential of these biobased materials, but economic feasibility is the major concern for its practical real time application. The plant flour–based materials and other ingredients are much costlier compared to the synthetic plastic-based ingredients. Anand et al. [81] recently studied the cost analysis and feasibility of edible coffee cup, and they reported that the average cost of reusable plastic cups is about 0.05 euro, while the paper-based one is three times higher (0.16 euro), and on the other hand, the PLA-based biodegradable cups average cost is 0.17 euro. Now, in comparison with this, the average cost of all the edible cups is 2.56 euro which is very much higher than the coffee itself. In another report, it has been shown that the cost of plastic cutlery is about 0.04 dollars, while the biodegradable cutlery cost was only 0.057 dollars which is 43% lower than the synthetic counterpart (https://open.library.ubc.ca/soa/cIRcle/collections/undergraduateresearch/18861/items/1.0108511). On the other side in another recent study, the cost of different edible cutlery was compared with edible cutlery compared with plastic one [82]. This report showed that the cost of plastic cutlery is 0.3 Rs. per plate, while the edible one is 3 Rs. per plate, and leaf/bamboo-based edible cutlery is 15 Rs. per plate, and in case of husk, it is 500 Rs. per plate. So, from this study, it is clear that the cost of the edible tableware is one of the major challenges for its practical application and this needs to reduce to make economically feasible. The valorization of agroindustrial waste in producing sustainable packaging could be an effective approaching in reducing the cost of the edible cutlery [83].

## 8. Conclusion and Future Perspectives

Edible cutlery is a biodegradable, sustainable, and healthy option, and thus, one of the best alternatives of synthetic and toxic plastic made cutlery. The edible cutlery can be prepared using nontoxic and ready to available materials like plant-based flour. Plant-based flours such as millet, rice, and wheat are commonly utilized ingredient to developed edible cutlery. The biobased edible cutlery can be further fortified/functionalized by adding natural antioxidant and antimicrobial agent. The incorporation of functional ingredient like onion peel powder, grape seed extract, ginseng powder, beetroot extract, hibiscus powder, and mosambi peel could be useful to add nutraceuticals to the edible cutlery. The fortification and functionalization of edible cutlery could be useful for the preservation as well as for health benefits. Moreover, the edible plateware can be prepared by simple mixing and baking method with employing oven, and the preparation process is hassle-free, short time required and can be developed without using high-end techniques. The properties of the edible cutlery are suitable to keep daily used food items and make food healthy and nutrition. One of the concerns related to edible cutlery is its cost which is comparatively higher than single-use plastics. In addition, the limited availability in the market and shorter durability are other concerns for the customers to purchase edible cutlery. At the end of the meal, edible cutlery can be eaten or disposed of; either way, it is beneficial without causing any harm to our health or environment. Therefore, the biobased edible tableware and cutlery can be a good alternative to the plastic-based cutlery, but still it is in the early stages and there are lot of development and research work to be done for further sustainable development in this field of research.

## Figures and Tables

**Table 1:** Physicochemical parameters and characterization analysis of different types of plant flour–based edible tableware.

Ingredient used in edible tableware	Physicochemical parameters	Characterization results	References
Cutlery items using soy protein isolates and crude MGSF	Color values and mechanical properties	• Physical and mechanical properties are retained by adding MGSF	[48]
	Water absorption, degree of swelling, solubility, and microstructure	• Water resistance properties and hue value improved in the presence of MGSF	
		• SEM analysis showed uniform distribution of MGSF without any crack on the cutlery	

Wheat flour, millet flour, xanthan gum, and grape seed flour-based edible spoon	Texture analysis, total phenolic content, and antioxidant activity	• The hardness of the spoon was enhanced in the presence of the binder (xanthan gum)	[49]
		• The functional properties (antioxidant activity and total phenolic content) of the spoon were increased due to the presence of grape seed flour	

Edible straw using all-purpose flour (rice, ragi, and wheat), yeast, and oil	Flexural strength test, tensile test, moisture absorption test, biodegradability test, material properties (yield strength, Young's modulus, Poisson's ratio)	• The edible straw material showed excellent baking properties and biodegradable properties	[50]
		• The moisture absorption, strength, and other properties were not much influenced	

Optimization of composition for the preparation of edible cutlery using response surface methodology (RSM)	Water absorption capacity hardness, fracturability, springiness, cohesiveness, gumminess, chewiness, and resilience	• Barnyard millet and wheat flour were found as excellent ingredient to make the cutleries	[51]
		• The optimized sample of biodegradable spoon withstands in cold water and hot water for 25 and 15 min, respectively, and degraded within 5 days	

Edible cutlery (spoon, fork, and bowl) using wheat flour, rice flour, sorghum flour, sugar, butter, salt,and vanilla essence	Proximate analysis, sensory analysis, biodegradability test (soil burial test), water holding capacity, and oil binding capacity	• Edible cutlery items using 40 g of rice flour, 30 g of sorghum flour, and 30 g of wheat flour along with other ingredients showed highest solvent retention capacity values, high tensile strength, and good sensory value	[52]
		• Based on the soil burial test, the developed cutlery items were degraded within 5–7 days	

Edible spoon and fork prepared by taking sorghum flour, wheat flour, and rice	Soil burial test and water absorption test	• Sample 2 (150 g of sorghum flour, 100 g of wheat and rice flour, 1 cup of water) showed higher strength test and resistive to hot water because of the content of high percentage of sorghum	[53]
		• All the samples did not show much difference in biodegradability test	
		• Water absorption percentage is found to be high in Sample 1 having wheat flour (150 g) and rice, sorghum flour (100 g)	

Edible cutlery (bowl and spoon) using rice flour, millet flour, wheat flour, jaggery, and banana blossom paste	Proximate analysis, water absorption percentage, soil burial test, and sensory evaluation (9-point hedonic scale)	• Sample 3 (40 g of rice flour, 20 g of millet flour, 30 g of wheat flour, 10 g of banana blossom powder, 15 g of jaggery, 1.5 g of oil, and 0.35 g of salt) showed the best proximate properties, water absorption capacity, and organoleptic properties	[54]

Edible cutlery by using moringa leaves powder, foxtail millet, finger millet, wheat, and rice	Moisture content, drying rate, moisture ratio	• All the drying characteristics of the edible spoon with respect to time were analyzed and found to be decreased	[55]

Edible cutlery spoon using *Withania somnifera* root powder, wheat flour, ragi flour, and sorghum flour	Sensory analysis, proximate analysis (moisture content, ash content, fat content, crude fiber content, protein), water absorption capacity, soil burial test, texture analysis, and antioxidant activity	• Based on the characteristics analysis of edible spoon, it was found to be very nutritious, tasty, healthy, and environmentally	[31]

Colored edible spoon using beetroot extract, spinach extract, and jamun extract along with sorghum flour, and wheat and rice flour	Proximate analysis, texture analysis, color analysis, and soil burial test	• The color analysis of different samples was found to be different based on the addition of different extracts which is obviously owing to the difference of the added extract	[56]
		• All the samples were decayed within 5–7 days indicating good biodegradability	

Edible cutlery using sorghum flour, wheat flour, and rice flour	Water absorption test and soil burial test	• Good result on water absorption test and soil burial test	[57]
		• The expense of made edible cutleries is analyzed and compared with plastic cutleries	

Nonwheat edible spoon by using raw cassava and saba banana	Water absorption test, acceptability evaluation, and shelf-life study	• Out of 5 formulations, formulations 1, 2, and 3 showed a little crumby texture, while formulations 4 and 5 showed firm and little sticky texture	[58]
		• Water absorption capacity of all formulated edible spoon is studied from 0 to 30 min and results indicated the absorption of water with increasing time duration as a result the spoon was soggy	

Ragi, refined wheat flour, and BSG-based edible tableware	Water and oil absorption, antioxidant, texture analysis, and biodegradation test	• The addition of BSG meaningfully reduced the water and oil absorption and the edible tableware was soggy after half an hour	[12]
		• The antioxidant activity and hardness of the tableware were enhanced significantly in the presence of BSG	
		• Moreover, the soil burial test showed that the edible bowl product was biodegradable	

Mosambi peel and sago powder, gum Arabic-based edible spoon	Moisture, ash, fat, protein content, water absorption, sensory analysis, organoleptic properties, and biodegradation test	• The moisture content of the 1:1 sago and peel combination-based edible spoon was lower than that of the control	[37]
		• The fat and protein content were also higher in 1:1 sago and peel combination	
		• The spoon shows excellent biodegradability in soil burial test	

Pine needles and paddy straw along with binding agent (carboxymethyl cellulose (CMC), potato starch, and gum acacia), and polylactic acid coating	Swelling, water and oil solubility, biodegradability, antimicrobial activity, water vapor permeability test, and mechanical properties	• Biodegradable cup, bowls, and table ware were prepared using paddy straw and pine needle	[22]
		• In case of CMC as binder, water solubility, oil solubility, and water absorption were less compared with starch and gum acacia	
		• The coated tableware showed better water vapor barrier and mechanical properties compared to uncoated counterpart	
		• The developed tableware showed good biodegradability and also some antibacterial activity toward *Staphylococcus* and *Pseudomonas* strain	

**Table 2:** Composition, mechanical properties, and biodegradability of different plant flour–based edible tableware.

Composition	Conditions	Mechanical properties	Biodegradability	References
Rice flour and rice bran	150°C + 7 min	Impact strength: 0.32 ± 0.0 kJ/m^2^ Flexural strength: 1.17 ± 0.05	Biodegraded in 15 days	[61]
Soy protein isolate + morning glory stem fiber	160°C + 5 min	Flexural strength 1619.2 ± 115.1 MPa	Highly biodegradable	[38]
Banana stem flour + sugarcane leaves + water hyacinth	—	Tensile strength: 6.23 MPa and elongation: 10.09%	Biodegraded in 20 days	[71]
Kombucha cellulose + tannery waste gelatin	—	Tensile strength of 47.7 MPa and a flexural strength of 117.27 MPa	Highly degradable and recycled as biofertilizer	[72]
Flax seed cake + wheat flour	150°C + 12 min	Strength test: 7.71 ± 0.54 MPa	Highly degradable	[73]
Mosambi peel and sago powder	80°C + 2 h	—	40%–60% biodegradability in 12 days	[37]
Sorghum and flower powder	—	Hardness increases in the presence of hibiscus and rose flower powder from 34 N to 53 and 59 N, respectively, while elastic force decreased	Spoon degraded in soil with 2 weeks	[74]
Sorghum and flower powder	80°C for 7 min at 1500 psi	Hardness and elastic force of flower containing bowl increased	> 95% degradation of bowl on 12 days in soil	[75]
Kodo millet, hibiscus powder, and guar gum	—	Tensile strength and flexural strength were slightly reduced from 53 to 47 MPa and 62 and 57 MPa, respectively	—	[76]
Rice, ragi, wheat flour, and yeast	180°C + 30 min	Stiffness increased from 20 to 44 MPa in the presence of yeast	—	[77]
Wheat flour, millet smooth flour, and grape flour	240°C + 10 min	Hardness increased with increase in temperature and xanthan concentration without adding oil	—	[55]
Finger millet, brewer spent grain	180°C + 30 min	Increase the concentration of brewer spent grain increased the hardness	Observed 30% weight loss in 10 weeks when buried in soil for degradation	[12]
Wheat flour, finger millet, sorghum flour, and Indian ginseng roots	100–150°C + 25 min	Hardness: 410 ± 0.81 N Fracturability: 1.8 ± 0.02 mm	—	[31]

## Data Availability

Data sharing is not applicable to this article as no new data were created or analyzed in this study.

## References

[1] Hasanzadeh R., Mojaver P., Azdast T., Chitsaz A., Park C. B. (2022). Low-Emission and Energetically Efficient Co-Gasification of Coal by Incorporating Plastic Waste: A Modeling Study. *Chemosphere*.

[2] Cera A., Cesarini G., Scalici M. (2020). Microplastics in Freshwater: What is the News From the World?. *Diversity*.

[3] Gupta V., Biswas D., Roy S. (2022). A Comprehensive Review of Biodegradable Polymer-Based Films and Coatings and Their Food Packaging Applications. *Materials*.

[4] Navarre N., Mogollón J. M., Tukker A., Barbarossa V. (2022). Recycled Plastic Packaging From the Dutch Food Sector Pollutes Asian Oceans. *Resources, Conservation and Recycling*.

[5] Smith M., Love D. C., Rochman C. M., Neff R. A. (2018). Microplastics in Seafood and the Implications for Human Health. *Current Environmental Health Reports*.

[6] Tran T. V., Jalil A. A., Nguyen T. M., Nguyen T. T. T., Nabgan W., Nguyen D. T. C. (2023). A Review on the Occurrence, Analytical Methods, and Impact of Microplastics in the Environment. *Environmental Toxicology and Pharmacology*.

[7] Boran H., Terzi S. (2019). Bis (2-Ethylhexyl) Phthalate Induces DNA Strand Breaks and Gene Expression Alterations in Larval Zebrafish *Danio rerio*. *Toxicology and Industrial Health*.

[8] Walker T. R., McGuinty E., Charlebois S., Music J. (2021). Single-Use Plastic Packaging in the Canadian Food Industry: Consumer Behavior and Perceptions. *Humanities and Social Sciences Communications*.

[9] Natarajan N., Vasudevan M., Vivekk Velusamy V., Selvaraj M. (2019). Eco-Friendly and Edible Waste Cutlery for Sustainable Environment. *International Journal of Engineering and Advanced Technology*.

[10] Michel C., Velasco C., Spence C. (2015). Cutlery Matters: Heavy Cutlery Enhances Diners’ Enjoyment of the Food Served in a Realistic Dining Environment. *Flavour*.

[11] Zhuo Y., He J., Li W., Deng J., Lin Q. (2023). A Review on Takeaway Packaging Waste: Types, Ecological Impact, and Disposal Route. *Environmental Pollution*.

[12] Nehra A., Biswas D., Roy S. (2022). Fabrication of Brewer’s Spent Grain Fortified Bio-Based Edible Bowls: A Promising Alternative to Plastic Containers. *Biomass Conversion and Biorefinery*.

[13] Vignesh P., Srinivasan S., Sundari S. M., Eswar S. K. (2023). An Investigation on MX/G/1 Queuing Model of Interrupted Services in the Manufacturing of Edible Cutlery Process. *International Journal of Mathematical Modelling and Numerical Optimisation*.

[14] Gong X., Shi G., Zou D. (2023). Micro-and Nano-Plastics Pollution and its Potential Remediation Pathway by Phytoremediation. *Planta*.

[15] Schmaltz E., Melvin E. C., Diana Z. (2020). Plastic Pollution Solutions: Emerging Technologies to Prevent and Collect Marine Plastic Pollution. *Environment International*.

[16] Manu M. K., Luo L., Kumar R. (2023). A Review on Mechanistic Understanding of Microplastic Pollution on the Performance of Anaerobic Digestion. *Environmental Pollution*.

[17] Schwabl P. (2020). Microplastics in Hot Water. *Nature Food*.

[18] Nguyen M. K., Lin C., Nguyen H. L. (2023). Emergence of Microplastics in the Aquatic Ecosystem and Their Potential Effects on Health Risks: The Insights Into Vietnam. *Journal of Environmental Management*.

[19] Rajvanshi J., Sogani M., Kumar A. (2023). Perceiving Biobased Plastics as an Alternative and Innovative Solution to Combat Plastic Pollution for a Circular Economy. *Science of the Total Environment*.

[20] Walker T. R., Fequet L. (2023). Current Trends of Unsustainable Plastic Production and Micro (Nano) Plastic Pollution. *TrAC, Trends in Analytical Chemistry*.

[21] Brown E., MacDonald A., Allen S., Allen D. (2023). The Potential for a Plastic Recycling Facility to Release Microplastic Pollution and Possible Filtration Remediation Effectiveness. *Journal of Hazardous Materials Advances*.

[22] Gupta A., Singh G., Ghosh P., Arora K., Sharma S. (2023). Development of Biodegradable Tableware From Novel Combination of Paddy Straw and Pine Needles: A Potential Alternative Against Plastic Cutlery. *Journal of Environmental Chemical Engineering*.

[23] Kumar S., Libertin A., Prakash A., Manikandan M., Sharathbabu S. (2024). Study on the Different Materials for Making Edible Plates for Sustainable Environment. *AIP Conference Proceedings*.

[24] Patil H. N., Sinhal P. (2018). A Study on Edible Cutlery: An Alternative for Conventional Ones. *Atithya: A Journal of Hospitality*.

[25] Priyadarshi R., Purohit S. D., Roy S., Ghosh T., Rhim J.-W., Han S. S. (2022). Antiviral Biodegradable Food Packaging and Edible Coating Materials in the COVID-19 Era: A Mini-Review. *Coatings*.

[26] Roy T. R., Morya S. (2022). Edible Cutlery: An Eco-Friendly Replacement for Plastic Cutlery. *Journal of Applied and Natural Science*.

[27] Sofi Dinesh K. G., Mitra S., Sisodia G. S., Ogunmokun O. A., Abichandani Y., Kumar P. (2024). Measuring the Consumer’s Choice for Edible Cutlery: A Sustainable Lifestyle Perspective. *Journal of Infrastructure, Policy and Development*.

[28] Reddy B. D. (2016). *Bakeys: You Can Use and Eat This Innovative Cutlery*.

[29] Das S. (2016). *How to Save the Planet, A Spoon at a Time*.

[30] Namratha B., Gaonkar S. L. (2024). Edible Cutlery: A Tenable Solution to the Plastic Menace, Bolstering the Global Economy. *Remediation of Plastic and Microplastic Waste*.

[31] Hazra S., Sontakke M. (2023). Process Development and Quality Evaluation Edible Cutlery Spoons Supplemented With *Withania Somnifera* Root Powder. *The Pharma Innovation Journal*.

[32] seyyedi S. r., Kowsari E., Ramakrishna S., Gheibi M., Chinnappan A. (2023). Marine Plastics, Circular Economy, and Artificial Intelligence: A Comprehensive Review of Challenges, Solutions, and Policies. *Journal of Environmental Management*.

[33] Kwon G., Cho D. W., Park J., Bhatnagar A., Song H. (2023). A Review of Plastic Pollution and Their Treatment Technology: A Circular Economy Platform by Thermochemical Pathway. *Chemical Engineering Journal*.

[34] Schug T. T., Birnbaum L. S. (2014). Human Health Effects of Bisphenol A. Toxicants in Food Packaging and Household Plastics. *Exposure and Health Risks to Consumers*.

[35] Mukherjee K., Raju A. (2023). Edible Cutlery–A Prototype to Combat Malnutrition and Plastic Waste Management. *Asian Journal of Biological and Life Sciences*.

[36] Nweze C. C., Nebechukwu E. W., Bawa M. Y. (2021). Dietary Fiber and Risk of Coronary Heart Diseases. *GSC Advanced Research and Reviews*.

[37] Siddiqui B., Ahmad A., Yousuf O., Younis K. (2023). Exploring the Potential of Mosambi Peel and Sago Powder in Developing Edible Spoons. *Sustainable Food Technology*.

[38] Choeybundit W., Shiekh K. A., Rachtanapun P., Tongdeesoontorn W. (2022). Fabrication of Edible and Biodegradable Cutlery From Morning Glory (Ipomoea Aquatic) Stem Fiber-Reinforced Onto Soy Protein Isolate. *Heliyon*.

[39] Dybka-Stępień K., Antolak H., Kmiotek M., Piechota D., Koziróg A. (2021). Disposable Food Packaging and Serving Materials—Trends and Biodegradability. *Polymers*.

[40] Rashid M. S. (2019). *Edible Cutleries as Sustainable Substitute for Plastic Cutleries*.

[41] Kaur J., Gunjal M., Rasane P. (2022). Edible Packaging: An Overview. *Edible Food Packaging: Applications, Innovations and Sustainability*.

[42] Sazeli Z. A., Zailani A., Tajudin I., Alif H. (2021). Biodegradable Cup as A Substitute for Single Use Plastic. *Multidisciplinary Applied Research and Innovation*.

[43] Kumbhar V., Masali P. (2020). Biodegradable Cutlery Using Moringapod Husk: An Alternative to Conventional Plastic Cutlery. *International Journal of Innovative Science and Research Technology*.

[44] Faustino M., Veiga M., Sousa P., Costa E. M., Silva S., Pintado M. (2019). Agro-Food Byproducts as a New Source of Natural Food Additives. *Molecules*.

[45] Gustafsson J., Landberg M., Bátori V., Åkesson D., Taherzadeh M. J., Zamani A. (2019). Development of Bio-Based Films and 3D Objects From Apple Pomace. *Polymers*.

[46] Buxoo S., Jeetah P. (2020). Feasibility of Producing Biodegradable Disposable Paper Cup From Pineapple Peels, Orange Peels and Mauritian Hemp Leaves With Beeswax Coating. *SN Applied Sciences*.

[47] Rajeshkumar G., Arvindh Seshadri S., Devnani G. L. (2021). Environment Friendly, Renewable and Sustainable Poly Lactic Acid (PLA) Based Natural Fiber Reinforced Composites–A Comprehensive Review. *Journal of Cleaner Production*.

[48] Chowdhury G. R., Dutta S., Pal N., Mitra A. (2021). Edible Cutlery: Futuristic Dining to Functional Sustenance. *Parana Journal of Science and Education*.

[49] Rajendran S. P., Saravanan A., Namachivayam G. K., Jambunathan J., Ramachandran G. (2020). Optimization of Composition for Preparation of Edible Cutlery Using Response Surface Methodology (RSM). *AIP Conference Proceedings*.

[50] Nishihara Y., Magashi Y. K. (2021). Fabrication of Shape-Changing Edible Structures by Extrusion-Based Printing and Baking. *Creativity and Cognition*.

[51] Molu K. R., Aneena E. R., Panjikkaran S. T. Effect of Finger Millet (Eleusine Coracana) Flour and Maize Flour on Nutritional, Texture and Physico-Chemical Qualities of Edible Dessert Cup.

[52] Putri A. K., Widayat H. P., Sulaiman M. I., Yusup E. M., Indarti E. (2025). Production of Edible Straw Based on Banana Flour and Breadfruit Flour Binding With Tapioca Starch. *IOP Conference Series: Earth and Environmental Science*.

[53] Senthilkumar P., Shankar S., Sivananda Moorthy R., Harirajan T., Kumar L. *Formulation and Quality Assessment of Biodegradable Edible Cutlery With Banana and Quinoa Integration*.

[54] Banh Q. N., To V. T., Luu M. M., Nguyen H. D., Dong V.-K. Optimization of Durability of Edible Spoon Using Design of Experiment Method.

[55] Dordevic D., Necasova L., Antonic B., Jancikova S., Tremlová B. (2021). Plastic Cutlery Alternative: Case Study With Biodegradable Spoons. *Foods*.

[56] Roy S., Ezati P., Rhim J.-W. (2022). Fabrication of Antioxidant and Antimicrobial Pullulan/Gelatin Films Integrated With Grape Seed Extract and Sulfur Nanoparticles. *ACS Applied Bio Materials*.

[57] Thagunna B., Shrestha G., Karki R., Baral K., Kaur J. (2023). Development and Quality Evaluation of Biodegradable Edible Cutlery: A Replacement for a Conventional One. *Asian Journal of Pharmaceutical and Clinical Research*.

[58] Kabir M. H., Hamidon N. (2021). A Study of Edible Cutleries by Using Sorghum Flour. *Progress in Engineering Application and Technology*.

[59] Boro M., Devi R. J., Sharma L. S. (2020). Biodegradable Cluteries and Tablewares as Substitute for Plastic: An Exploratory Study on Green Solutions. *International Journal of Research and Scientific Innovation (IJRSI)*.

[60] Rishi P., Zade T., Abhiraj P., Pandey V. S., Roy S. (2024). Exploring the Potential of Ragi, Wheat, and Rice Flours as a Sustainable Solution for Edible Bowl: A Suitable Substitution for Disposable Plastic Bowl. *Food and Humanity*.

[61] Yodkum T., Pajareon S., Yokesahachart C. (2024). Effect of Defatted Rice Bran Content on Physicochemical and Sensory Properties of Edible Cutlery Made From Rice Flour Green Composites Using Compression Molding. *Journal of Current Science and Technology*.

[62] Kabir M. H., Hamidon N., Awang M., Rahman M. A. A., Adnan S. H. (2021). An Edible Cutleries Using Green Materials: Sorghum Flour. *Green Infrastructure: Materials and Applications*.

[63] Evoware (2021). *Seaweed-Based Packaging*.

[64] Iqbal B., Raza R., Khan N., Siddiqui K. A. (2022). Bio-friendly Edible Cutlery-An Effective Alternative to Plastic Disposable Cutlery. *Journal of Research*.

[65] Shabaana M., Firdouse T. F., Prabha P. H. (2021). Development and Quality Evaluation of Eco Friendly Moringa Oleifera Leave Powder Incorporated Edible Cutlery. *International Journal of Advances in Engineering and Management*.

[66] Sindhu K., Swamy R., Prashanthi M. (2023). Enrichment of Edible Spoons With Natural Colours. *The Pharma Innovation Journal*.

[67] Patil T. D., Bisht S., Meshram B. P., Gaikwad K. K. (2025). A Review on Emerging Trends and Developments in Edible Drinking Straws for Food and Beverage Applications. *Trends in Food Science & Technology*.

[68] Vyshali P., Serena P. B. (2022). Development of an Edible and Biodegradable Tableware Using Fruit Wastes-An Alternative to Plastic Tableware. *International Journal of Food and Nutrition Science*.

[69] Habla F. A., Estrada J. P., Fulgar E. D., Hamos E. D. P., Escopete A. J. (2023). Development of Non-Wheat Edible Spoon. *Sorsogon Multidisciplinary Research Journal*.

[70] Kabir M. H., Hamidon N., Awang M., Rahman M. A. A., Adnan S. H. (2022). An Edible Cutleries Using Green Materials: Sorghum Flour. *Green Infrastructure*.

[71] Harikrishnan M. P., Raghunathan R., Warrier A. S. (2023). Reinforced Water Hyacinth Based Biodegradable Cutlery: Green Alternative to Single-Use Plastics. *Food Packaging and Shelf Life*.

[72] Muralidharan V., Jebathomas C. R. T., Sundaramoorthy S., Madhan B., Palanivel S. (2024). Preparation and Evaluation of Novel Biodegradable Kombucha Cellulose-Based Multi-Layered Composite Tableware. *Industrial Crops and Products*.

[73] Andrejko D., Blicharz-Kania A. (2024). An Assessment of the Strength and Physical Properties of Edible Tableware From Flax Seed and Flaxseed Cake. *Materials*.

[74] Manivel D., Paramasivam R. (2024). Sorghum Spoons Enriched With Selected Edible Flowers: A Sustainable Alternative to Conventional Cutlery in the Food and Tourism Sectors. *Biomass Conversion and Biorefinery*.

[75] Manivel D., Paramasivam R., Roy S., Optimizing S. (2025). Optimizing Edible Sorghum Bowls: Effects of Roasting and Edible Flower Powder Enhancement on Technological, Nutritional, Antioxidant, and Functional Properties. *International Journal of Food Science*.

[76] Meshram B. P., Jain P., Gaikwad K. K. (2025). Innovative Development of Kodo Millet (Paspalum Scrobiculatum)-Based Functional Edible Cups Modified With Hibiscus Powder and Guar Gum: An Eco-Efficient Resource Utilization. *ACS Food Science & Technology*.

[77] Yavagal P. S., Kulkarni P. A., Patil N. M. (2020). Cleaner Production of Edible Straw as Replacement for Thermoset Plastic. *Materials Today: Proceedings*.

[78] Narvekar S. (2022). *Review of Innovations in the Use of Edible Containers and Cutlery*.

[79] Brownlee A., Li C., Lo M. (2013). Life Cycle Assessment: Aspenware Biodegradable Cutlery.

[80] Pandya V. S., Fiorillo L., Kalpe S. (2023). Veganism and Oral Health—An Overview Through the Perspective. *European Journal of General Dentistry*.

[81] Anand K., Martinez Arce A., Bishop G., Styles D., Fitzpatrick C. (2024). A Tasty Solution to Packaging Waste? Life Cycle Assessment of Edible Coffee Cups. *Resources, Conservation and Recycling*.

[82] Gupta M., Sanghi D. (2023). *Edible Cutlery: An Emerging Sustainable Approach Towards a Healthy Future*.

[83] Jamwal V., Mittal A., Dhaundiyal A. (2024). Valorization of Agro-Industrial Waste in Composite Films for Sustainable Packaging Applications. *Materials Today: Proceedings*.

